# Viral Inactivation Impacts Microbiome Estimates in a Tissue-Specific Manner

**DOI:** 10.1128/mSystems.00674-21

**Published:** 2021-10-05

**Authors:** Alba Boix-Amorós, Enrica Piras, Kevin Bu, David Wallach, Matthew Stapylton, Ana Fernández-Sesma, Dolores Malaspina, Jose C. Clemente

**Affiliations:** a Department of Genetics and Genomic Sciences, Icahn School of Medicine at Mount Sinai, New York, New York, USA; b Precision Immunology Institute, Icahn School of Medicine at Mount Sinai, New York, New York, USA; c Department of Microbiology, Icahn School of Medicine at Mount Sinai. New York, New York, USA; d Department of Neuroscience, Icahn School of Medicine at Mount Sinai. New York, New York, USA; Princeton University

**Keywords:** 16S RNA, DNA sequencing, human microbiome, viral inactivation

## Abstract

The global emergence of novel pathogenic viruses presents an important challenge for research, as high biosafety levels are required to process samples. While inactivation of infectious agents facilitates the use of less stringent safety conditions, its effect on other biological entities of interest present in the sample is generally unknown. Here, we analyzed the effect of five inactivation methods (heat, ethanol, formaldehyde, psoralen, and TRIzol) on microbiome composition and diversity in samples collected from four different body sites (gut, nasal, oral, and skin) and compared them against untreated samples from the same tissues. We performed 16S rRNA gene sequencing and estimated abundance and diversity of bacterial taxa present in all samples. Nasal and skin samples were the most affected by inactivation, with ethanol and TRIzol inducing the largest changes in composition, and heat, formaldehyde, TRIzol, and psoralen inducing the largest changes in diversity. Oral and stool microbiomes were more robust to inactivation, with no significant changes in diversity and only moderate changes in composition. *Firmicutes* was the taxonomic group least affected by inactivation, while *Bacteroidetes* had a notable enrichment in nasal samples and moderate enrichment in fecal and oral samples. *Actinobacteria* were more notably depleted in fecal and skin samples, and *Proteobacteria* exhibited a more variable behavior depending on sample type and inactivation method. Overall, our results demonstrate that inactivation methods can alter the microbiome in a tissue-specific manner and that careful consideration should be given to the choice of method based on the sample type under study.

**IMPORTANCE** Understanding how viral infections impact and are modulated by the microbiome is an important problem in basic research but is also of high clinical relevance under the current pandemic. To facilitate the study of interactions between microbial communities and pathogenic viruses under safe conditions, the infectious agent is generally inactivated prior to processing samples. The effect of this inactivation process in the microbiome is, however, unknown. Further, it is unclear whether biases introduced by inactivation methods are dependent on the sample type under study. Estimating the magnitude and nature of the changes induced by different methods in samples collected from various body sites thus provides important information for current and future studies that require inactivation of pathogenic agents.

## INTRODUCTION

A homeostatic microbiome is essential at the physiological, immunological, and metabolic levels for human health, and alterations in its composition have been linked to several diseases, ranging from intestinal inflammatory conditions (1–3) to respiratory infections (4, 5), asthma and allergies (6–8), or even neurological disorders (9–11). Furthermore, growing evidence suggests that alterations in the human microbiome may also occur in response to viral infections (12–17). Bacteria can also play important roles during viral infection processes, ranging from offering protection against viral agents (18–22) to facilitating viral infections or participating in bacterial-viral coinfections (23–27). The continuous emergence of novel pathogenic viruses at a global scale, such as the H1N1 influenza A virus (28, 29), or the coronaviruses responsible for the severe acute respiratory syndrome (SARS-CoV) (30, 31), the Middle East respiratory syndrome (MERS-CoV) (32, 33), or the more recent SARS-CoV-2 causative of the current COVID-19 pandemic (34, 35), emphasizes the need to understand how microbial communities might be related to these pathogens and whether they modulate infection risk.

Working with highly infectious viral agents requires biosafety level 3 (BSL3) or 4 (BSL4) containment laboratories, which poses significant challenges for most researchers. Biosafety labs are generally limited to specialized research centers or hospitals and often cannot accommodate specific equipment, such as flow cytometers or microscopes, which are required for analyses. Viral inactivation methods allow the removal of highly infectious agents and facilitate the processing of samples in lower-level biosafety conditions following appropriate safety practices (28, 36, 37), thus expanding the analyses that can be performed on such samples. The 2019 SARS-CoV-2 pandemic, for example, has resulted in a widespread interest for researchers to study clinical samples from subjects with known or suspected COVID-19. These studies often require the use of effective viral inactivation methods that allow the manipulation of samples in different biosafety research environments. Several methods have been shown to effectively inactivate SARS-CoV-2 and have permitted the study of the virus in laboratories of different scientific backgrounds in an unprecedented global effort to unravel the virus pathogenesis and end the pandemic (38–41). Previous studies have shown that sample storage methods and, in particular, different temperatures, freeze-thaw cycles, and DNA preserving reagents, can impact microbial communities’ composition and stability (42–48) and that some microbial inactivation methods differentially preserve microbial nucleotides for subsequent PCR or immunoassay analysis (39, 44, 49). However, there is currently no evidence on the effect of viral inactivation methods in microbial composition and structure, which potentially represents a major source of biases in studies characterizing the microbiome of infected subjects (12, 16, 17, 50–53). It is therefore fundamental to quantify the effect of viral inactivation methods on different sample types to ensure the robustness of conclusions.

Here, we compared microbiome composition and diversity, as inferred from 16S rRNA amplicon sequencing, after treating samples representative of four different body sites (oral, nasal, skin, and stool) with five commonly used viral inactivation methods or reagents (heat killing at 56°C for 30 min, 75% ethanol, psoralen, 4%formaldehyde, and TRIzol) and compared them with untreated samples. Our results thus aim to identify inactivation methods that ensure the preservation of microbial communities across different body sites.

## RESULTS

### Microbial community composition is impacted by viral inactivation protocols.

**FIG 1:**
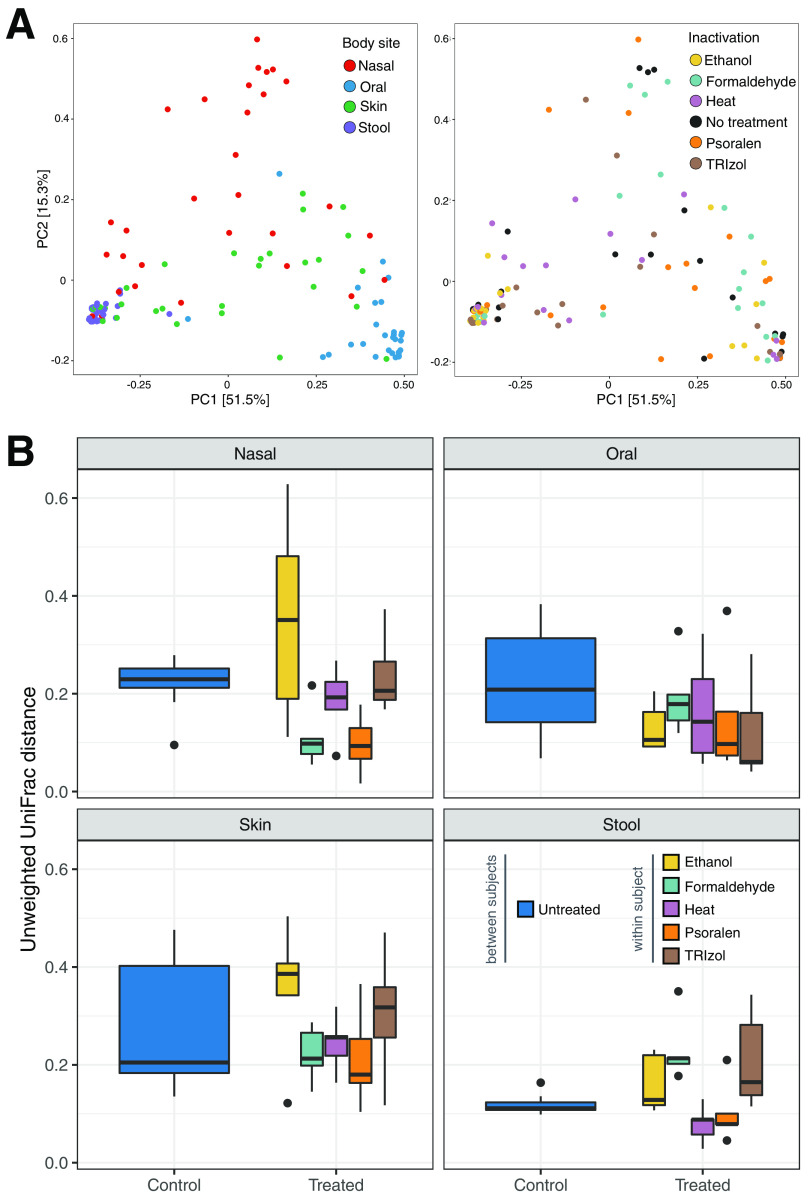
Impact of body site and inactivation treatments in microbiome composition. (A) PCoA plots based on weighted UniFrac distances, with samples colored by body site (left) and inactivation protocol (right). (B) Weighted UniFrac distances between untreated samples from different subjects and between untreated and inactivated samples from the same subjects for each inactivation method and body site.

To assess differences in bacterial community structure, we performed principal coordinate analysis (PCoA) based on weighted UniFrac distances. In the absence of inactivation (“no treatment”), samples clustered by body site (Kruskal-Wallis, *P* < 0.001) (Fig. S1 in the supplemental material), with dispersion of samples (variances) also differing significantly across body sites (permutation test, *P < *0.001), as previously reported (54). Stool and oral samples separated along the first principal coordinate, while nasal and skin samples were more scattered on the plot, indicative of a more variable microbiome composition. While samples separated primarily by body site, there were also significant changes associated with different inactivation protocols (Fig. 1A). These observations were confirmed by a distance-based permutational multivariate analysis of variance (adonis), which showed that body site and inactivation were both significantly associated with composition (*P = *0.001), although body site explained a larger percentage of the total variability in the data set (43% versus 7%).

10.1128/mSystems.00674-21.1 FIG S1 Differences in beta diversity across body sites in nontreated samples. PCoA plots based on weighted UniFrac show distances across different samples in the absence of viral inactivation. Samples are colored by body site. Download FIG S1, PDF file, 0.01 MB. Copyright © 2021 Boix-Amorós et al. 2021 Boix-Amorós et al. https://creativecommons.org/licenses/by/4.0/ This content is distributed under the terms of the Creative Commons Attribution 4.0 International license.

We next tested whether inactivation methods introduce changes in microbiome structure of less magnitude than those observed between untreated samples of different subjects. For each body site, we compared the distribution of UniFrac distances in untreated samples of different subjects (i.e., the intersubject distances) against the distribution of distances between the untreated and inactivated samples of each subject (the intrasubject distances) for each inactivation method (Fig. 1B). While the degree of change varied per body site and method, all inactivation protocols resulted in distances from the untreated sample that were not significantly different than those observed between untreated samples of different subjects (analysis of variance [ANOVA], *P* > 0.05). Oral and stool samples were, in general, the least affected by inactivation, while psoralen was the protocol that most consistently conserved the microbiome structure of the samples. Ethanol introduced particularly large changes in nasal and skin samples, while TRIzol disrupted more noticeably the microbiome of skin and stool samples. Overall, these results demonstrate that inactivation methods introduce notable changes to the microbiome across all body sites, of similar magnitude to those observed when comparing the microbial communities of different subjects.

### Viral inactivation induces shifts in alpha diversity.

**FIG 2:**
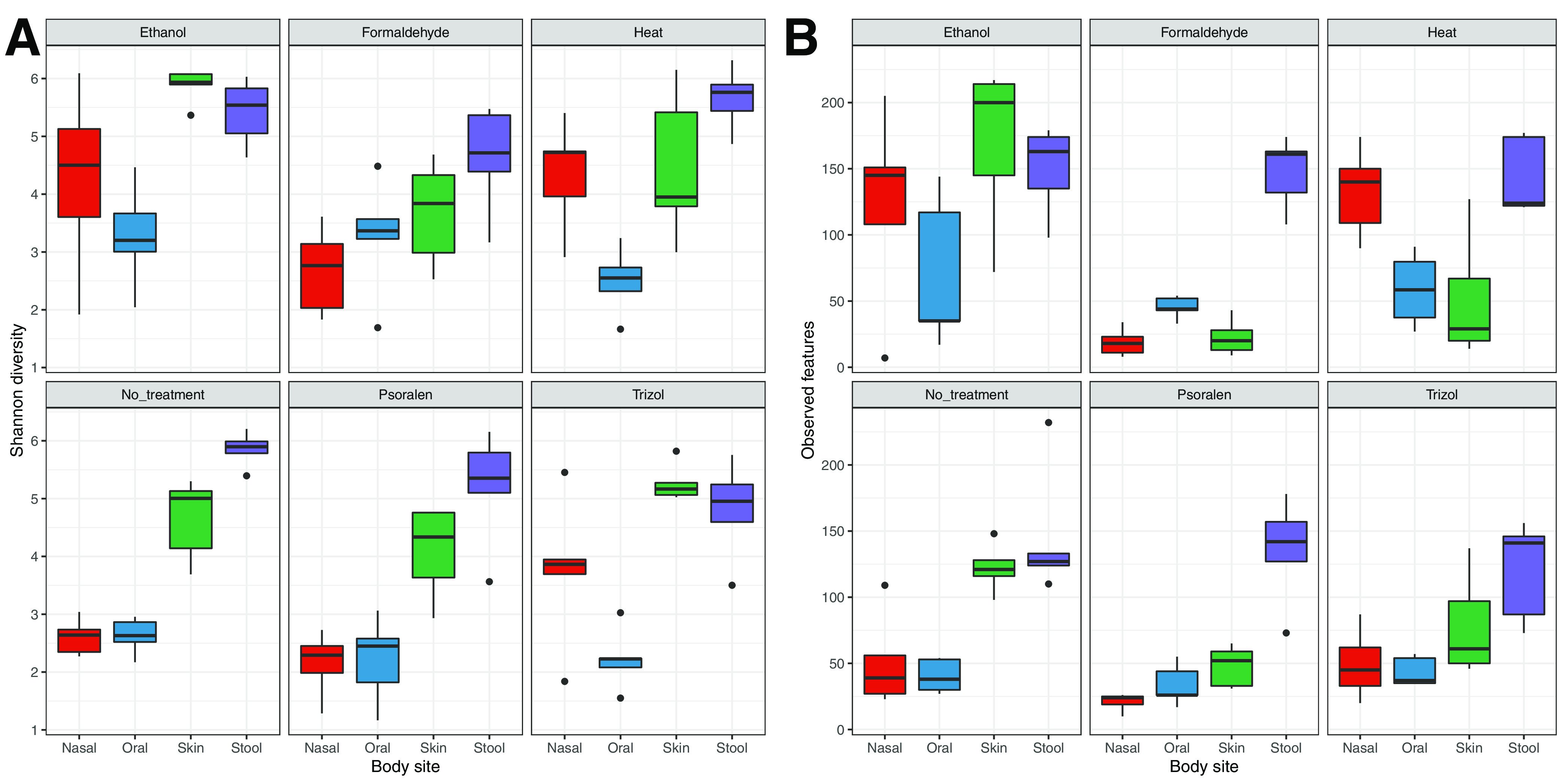
Microbial alpha diversity across inactivation treatments. (A) Observed number of features per inactivation method and body site. (B) Shannon diversity indices per inactivation method and body site. Samples appear colored by body site.

Microbial alpha diversity also varied across body sites, as observed in the nontreated samples (Kruskal-Wallis, observed features, *P* = 0.003; Shannon, *P* = 0.001). Both the number of observed features and Shannon indices varied significantly across body sites (Kruskal-Wallis, *P* = 1.1E^−8^ and *P* = 5.3E^−13^, respectively) and inactivation treatments (*P* = 0.002; *P* = 0.039) (Fig. S2). We further used generalized linear models to study the effect of inactivation treatments in alpha diversity when controlling for sample type and subject. Formaldehyde treatment reduced Shannon diversity in skin and stool samples (*P =* 0.02 and *P* = 0.01, respectively), while heat and TRIzol increased the diversity of nasal samples (*P* = 0.01; *P* = 0.03) (Fig. 2A). On the other hand, formaldehyde, heat, and psoralen treatments resulted in a decrease in the observed number of features in skin samples (*P* = 0.001, *P* = 0.008, and *P* = 0.03, respectively), while heat inactivation of nasal samples resulted in an increase in the observed features (*P* = 0.046) (Fig. 2B).

10.1128/mSystems.00674-21.2 FIG S2 Alpha diversity per inactivation treatment and body sites. Box plots show the observed number of features and Shannon diversity indices per sample type or inactivation treatment. Download FIG S2, PDF file, 0.04 MB. Copyright © 2021 Boix-Amorós et al. 2021 Boix-Amorós et al. https://creativecommons.org/licenses/by/4.0/ This content is distributed under the terms of the Creative Commons Attribution 4.0 International license.

### Changes in taxonomic profiles after inactivation.

**FIG 3:**
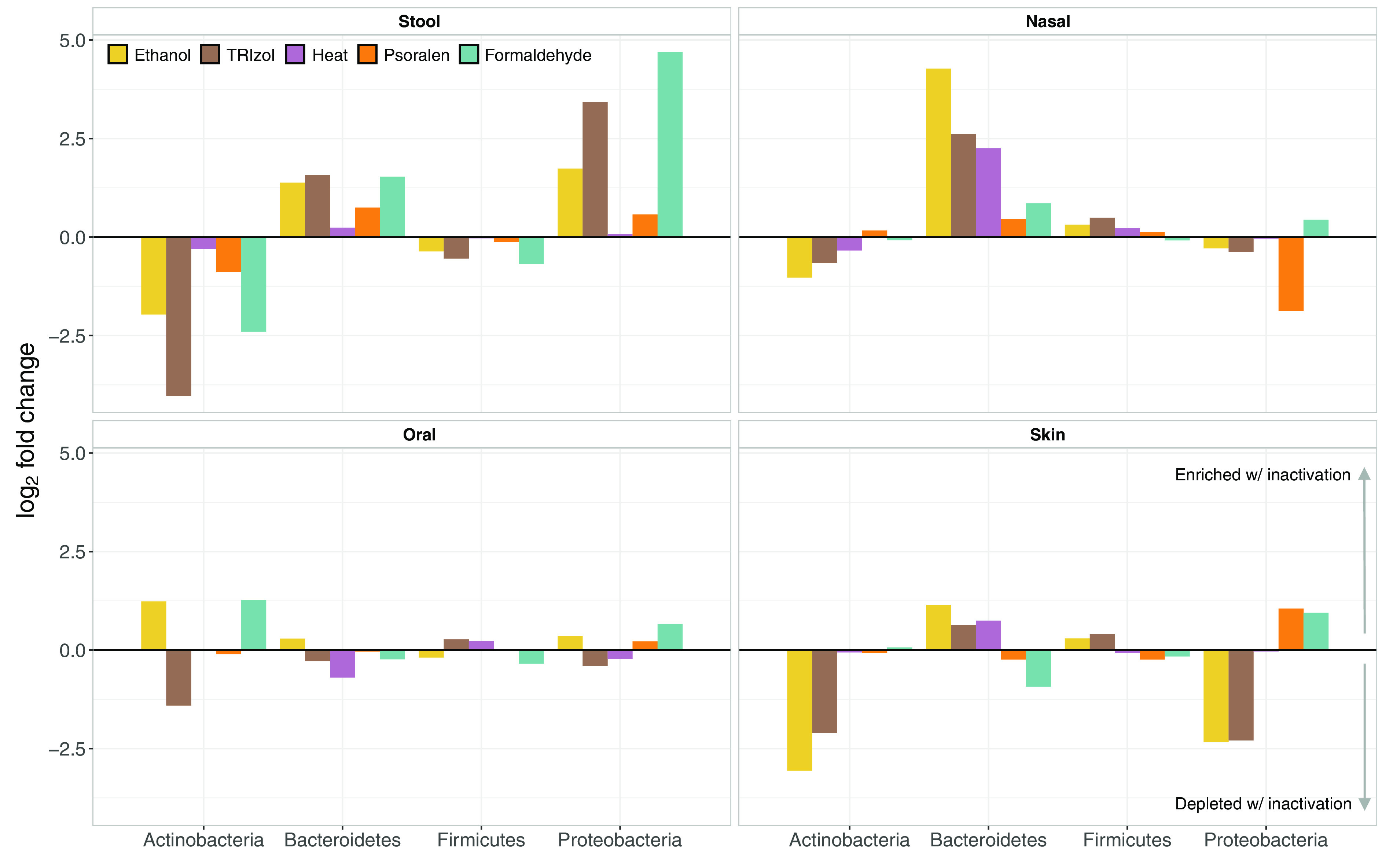
Enrichment and depletion across body sites and inactivation treatments for major bacterial phyla. Barplots represent the log_2_ fold change in bacterial relative abundances at the phylum level between each inactivation treatment and no treatment. Fold changes with positive values represent an increase in relative abundance after inactivation, while negative values indicate reduced relative abundances.

We next evaluated the effect of the inactivation methods on the taxonomic profiles of each sample type (Fig. S3). At the phylum level, stool samples were dominated by *Firmicutes* (mean ± standard deviation [SD], 70% ± 20%) and *Bacteroidetes* (25% ± 17%). Oral samples were dominated by *Firmicutes* (61% ± 24%) and *Proteobacteria* (30% ± 23%), and nasal and skin samples were dominated by *Firmicutes* (45% ± 18% and 65% ± 16%, respectively), *Actinobacteria* (38% ± 23% and 14% ± 11%, respectively) and *Proteobacteria* (11% ± 14% and 11% ± 13%, respectively). Taxonomic composition was differentially affected by inactivation treatment, depending on sample type (Fig. 3). While oral samples were generally stable and did not exhibit major changes with any method, all other sample types were impacted by the inactivation process. Across all samples, *Firmicutes* was the phylum least affected by inactivation treatments, while the effect on the other phyla varied in magnitude depending on the sample type. Stool samples were depleted of *Actinobacteria*, while abundances of *Proteobacteria* and, to a lesser extent, *Bacteroidetes*, were enriched across inactivation treatments. In this sample type, heat and psoralen introduced only small amounts of change. On the other hand, inactivation treatments led to a notable enrichment of *Bacteroidetes* in the nasal samples, particularly with ethanol. Other phyla were generally robust to inactivation treatments except the *Proteobacteria*, which was depleted by psoralen in these samples. Skin samples were mostly affected by ethanol and TRIzol, which depleted *Actinobacteria* and *Proteobacteria*, with moderate enrichment of the *Proteobacteria* (psoralen, formaldehyde) and the *Bacteroidetes* (ethanol, TRIzol, heat).

10.1128/mSystems.00674-21.3 FIG S3 Bacterial composition in microbiome samples depending on viral inactivation methods. Barplot shows average relative abundance of the bacterial taxa detected in nasal, oral, skin, and stool samples treated with different viral inactivation methods (ethanol, formaldehyde, heat, psoralen, TRIzol, or no treatment), as inferred by means of Illumina MiSeq sequencing of the 16S rRNA gene. Download FIG S3, PDF file, 0.03 MB. Copyright © 2021 Boix-Amorós et al. 2021 Boix-Amorós et al. https://creativecommons.org/licenses/by/4.0/ This content is distributed under the terms of the Creative Commons Attribution 4.0 International license.

### Differential enrichment analysis identifies tissue-specific effects of inactivation.

**FIG 4:**
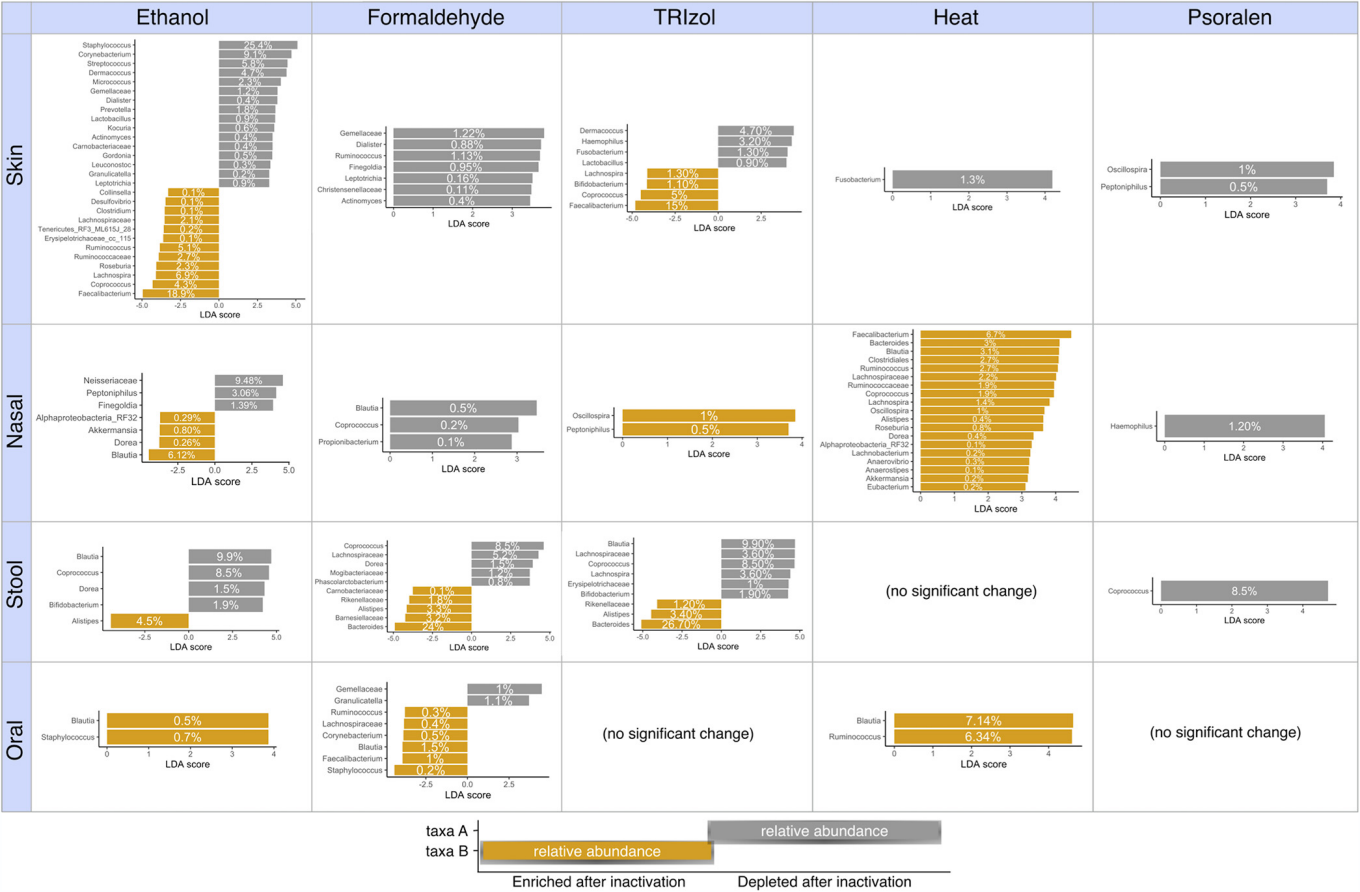
Bacterial taxa associated with viral inactivation treatments across body sites. Differentially abundant taxa enriched or depleted with each inactivation treatment and for each body site, as inferred by the LEfSe algorithm for biomarker discovery. The threshold for logarithmic discriminant analysis (LDA) score was 2, and significance was a *P* value of <0.05.

We further analyzed the effect of inactivation in microbial composition using differential enrichment analysis (55) to identify specific taxa that were significantly enriched or depleted at each body site and for each inactivation method (Fig. 4).

Overall, inactivation with ethanol and formaldehyde introduced the highest variability, as linear discriminant analysis effect size (LEfSe) analysis identified multiple differential taxa across all four body sites (Fig. S4). Across body sites, ethanol and formaldehyde induced changes in all sample types. TRIzol also introduced compositional differences in all sample types except for oral. Inactivation with heat resulted in moderate changes that were tissue dependent, and psoralen induced only a minimal reduction of bacterial abundances in the inactivated samples compared to no treatment (Fig. 4).

10.1128/mSystems.00674-21.4 FIG S4 Bacterial taxa associated with viral inactivation treatments. Differentially abundant taxa enriched or depleted with each inactivation treatment, as inferred by LEfSe. The threshold for logarithmic discriminant analysis (LDA) score was 2, and significance was a *P* value of <0.05. Download FIG S4, PDF file, 0.3 MB. Copyright © 2021 Boix-Amorós et al. 2021 Boix-Amorós et al. https://creativecommons.org/licenses/by/4.0/ This content is distributed under the terms of the Creative Commons Attribution 4.0 International license.

Skin and nasal samples had the highest number of differentially abundant taxa after inactivation. Skin samples were particularly sensitive to ethanol, as shown by a higher logarithmic discriminant analysis (LDA) effect size and a larger number of differentially abundant genera. TRIzol also increased the abundance of several taxa, including *Faecalibacterium*, *Coprococcus*, and *Lachnospira*, while some common skin microbes, such as Staphylococcus, *Corynebacterium*, and *Dermacoccus*, were depleted. Compared to formaldehyde, heat, and psoralen treatments, untreated skin samples also showed an enrichment of other low-abundance bacteria, including *Gemellaceae*, *Ruminococcus*, and *Fusobacterium*. Nasal samples were mostly affected by ethanol, TRIzol, and heat treatments, which resulted in an enrichment of *Blautia*, *Ruminococcus*, *Faecalibacterium*, and *Bacteroides*, among others. Ethanol also depleted *Neisseriaceae* and *Peptoniphilus*. In stool samples, ethanol enriched the genus *Alistipes*, while formaldehyde and TRIzol enriched mostly *Bacteroides*. On the other hand, all three treatments reduced the abundance of different fecal microorganisms, including *Blautia*, *Coprococcus*, and *Lachnospiraceae*, among others. Psoralen and heat preserved microbial communities in stool samples more robustly, with only *Coprococcus* being impacted by psoralen. Finally, oral samples showed the least number of taxa differentially enriched after inactivation. Formaldehyde treatment reduced the abundance of *Gemellaceae* and *Granulicatella* and increased the abundance of *Blautia* and *Faecalibacterium* (among others). Heat increased the abundance of *Blautia* and *Ruminococcus*, while TRIzol and psoralen treatments did not affect oral composition significantly.

To account for potential associations between viral inactivation methods and enrichment in bacterial contaminants commonly found in kits and other laboratory reagents, we compared the abundance of contaminant taxa (Table S1) across inactivation methods. Analysis of composition of microbiomes (ANCOM) (56) identified one family, *Bradyrhizobiaceae*, that was enriched in formaldehyde, heat, and TRIzol treatments (*W* = 67) (Fig. S5).

10.1128/mSystems.00674-21.5 FIG S5 Compositional differences across common laboratory reagent contaminants across inactivation methods. Volcano plot depicting results of ANCOM analysis. One taxon, *Bradyrhizobiaceae* (top right corner), was identified as differential across inactivation treatments (*W* = 67). Download FIG S5, PDF file, 0.03 MB. Copyright © 2021 Boix-Amorós et al. 2021 Boix-Amorós et al. https://creativecommons.org/licenses/by/4.0/ This content is distributed under the terms of the Creative Commons Attribution 4.0 International license.

10.1128/mSystems.00674-21.6 TABLE S1 List of contaminant taxa removed from the analysis. Download Table S1, XLSX file, 0.05 MB. Copyright © 2021 Boix-Amorós et al. 2021 Boix-Amorós et al. https://creativecommons.org/licenses/by/4.0/ This content is distributed under the terms of the Creative Commons Attribution 4.0 International license.

## DISCUSSION

Highly pathogenic viruses can only be handled in biocontainment BSL3 or BSL4 laboratories and must be rendered noninfectious before samples can be manipulated at lower biosafety levels. In this study, we aimed to quantify the effect of five commonly used inactivation methods (heat, ethanol, psoralen, TRIzol, and formaldehyde) in the microbiomes of samples collected from four human body sites. Because each individual harbors a unique microbiome (57, 58), inactivation methods that introduce changes lower than those observed between different subjects are desirable. By comparing the effect of these methods versus untreated samples, we estimated the magnitude of change introduced by each inactivation protocol and identified their biases across body sites. Overall, every method introduced compositional and diversity changes that differed across tissues (Table 1). Skin samples were the most impacted by inactivation and exhibited the highest levels of dispersion, followed by nasal samples. Oral samples, on the other hand, were mostly stable regardless of treatment, showing only minor differences compared to untreated samples. Despite having the lowest microbiome variability among the body sites under study, stool samples were also impacted by inactivation, particularly in the abundance of specific bacterial phyla. *Firmicutes* consistently had the smallest amount of change across inactivation methods. *Bacteroidetes* suffered minor changes in skin and oral samples, moderate in fecal, and high in nasal samples. *Actinobacteria* were highly depleted in fecal and skin samples (particularly with ethanol and TRIzol in the latter), moderately depleted in oral samples treated with ethanol or TRIzol, and mostly unchanged in nasal samples. Finally, *Proteobacteria* were highly impacted in fecal samples, highly depleted in nasal samples treated with psoralen, and highly depleted in skin samples treated with ethanol and TRIzol.

**TABLE 1:**
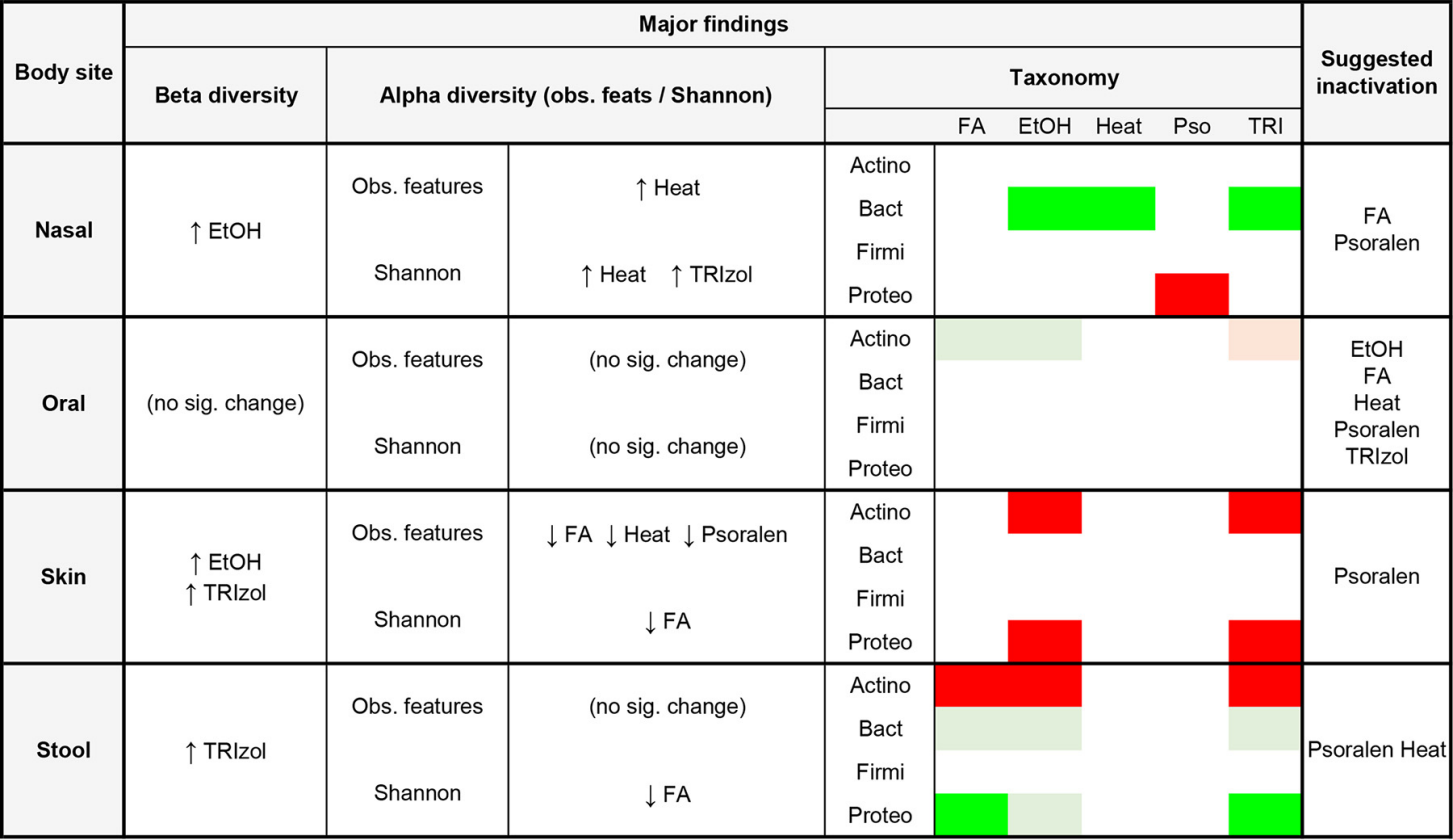
Summary of the effects of inactivation methods in microbial diversity estimates and taxonomy^*a*^

a Bright green, high enrichment; pale green, moderate enrichment; pale red, moderate depletion; bright red, high depletion.

Ethanol treatment introduced the largest amount of change across sample types. Alcohols can effectively inactivate a wide spectrum of bacteria, fungi, and viruses by disrupting cell membranes and denaturing proteins (59–61). At the same time, alcohols’ fixative properties preserve viral and bacterial structures and safely allow further downstream analysis (61–63). For this reason, ethanol has been commonly used as preserving agent in microbiome samples (64–66). Due to its low cost and accessibility, ethanol is an attractive option for viral inactivation. However, and to the best of our knowledge, the effect of inactivation using ethanol on microbiome composition and diversity has not been evaluated previously. Here, we used a 75% ethanol formulation (67–69) and found that the stability of bacterial communities was generally compromised by this treatment, particularly in skin and nasal samples. In skin samples, important commensals such as Staphylococcus or *Corynebacterium* were greatly depleted after ethanol treatment, while we observed an enrichment in bacteria such as *Faecalibacterium*, *Lachnospira*, or *Coprococcus*. In nasal samples, *Neisseriaceae* relative abundance was reduced, while that of *Blautia* increased. Ethanol inactivation resulted in moderate changes in stool samples and only introduced small changes in oral samples, in both cases affecting mostly bacterial taxa present at low abundance. The virucidal properties of ethanol are optimal when used at 60% to 80% concentration, as water facilitates protein denaturation (59, 70, 71). However, previous studies have shown that preserving microbiome samples with 70% ethanol can have detrimental effects on DNA yields, resulting in lower concentrations than freezing or other preservation methods (43, 65, 66).

TRIzol also introduced important changes in the microbiome of different body sites, particularly skin and stool. TRIzol is a widely used lysing solution that has been classically used for the cellular extraction of nucleic acids and proteins. The combined effect of phenol and guanidine isothiocyanate components of TRIzol disrupts cell membranes and denatures proteins, making this reagent effective at inactivating viruses (49, 72–74). Buffers containing TRIzol are commonly used for sample storage, as TRIzol also denatures DNase and RNase enzymes, thus protecting DNA and RNA from degradation (75). However, in our study, TRIzol treatment had important effects across samples from different body sites. Skin samples were especially affected by TRIzol, and, as we observed with ethanol, biases were notable in a wide variety of taxa. Stool and nasal samples had only moderate changes, while oral samples seemed to be robust to this treatment. In a recent study, incubation of samples containing SARS-CoV-2 with 10% TRIzol for 10 min was shown to reduce the levels of viral RNA detection by digital PCR (38). Further, it has been previously shown that microbiome samples stored in TRIzol had lower diversity than storage in phosphate-buffered saline (PBS), suggesting that this reagent may deplete part of the nucleic acids in the samples and thus may not be suitable for microbiome studies (75).

Formaldehyde resulted in a more heterogeneous set of results in our study. Formaldehyde is a fixative agent that is ubiquitously used in laboratory settings, including analysis of infected cells or microbial preparations for microscopy or flow cytometry. By cross-linking proteins, formaldehyde is able to inactivate cells while preserving their structural form, and it has been widely used in various concentrations for the inactivation of viruses (49, 73, 76–79). However, this reagent alters cellular structures and can render samples unusable for some molecular and/or immunological downstream analysis (80, 81). While nasal samples seemed to suffer only minimal effects, formaldehyde introduced moderate changes in oral, skin, and stool samples. Previous reports have shown partial bacterial, fungal, and viral inactivation with formaldehyde, likely due to inherent differences in microbial cell walls composition, i.e., peptidoglycan thickness (82–85). Therefore, it is possible that the effects of this reagent on the microbiome may differ depending on the precise bacteria composing each sample type.

Inactivation with psoralen had the least effect on microbial communities across body sites. Psoralen is a photoreactive, small compound of natural origin that has a wide range of pharmacologic applications. Psoralen can cross phospholipid bilayers and intercalate into nucleic acids and, upon exposure to UV (UV-A) radiation (PUVA), causes interstrand cross-linking and impedes replication. Psoralen effectively inactivates pathogens, including viral agents, while preserving structures and nucleic acids that allow for further antigenic and genomic analyses to be performed (37, 86–89). In our results, psoralen generally resulted in minimal changes compared to other inactivation treatments. Although PUVA is a common phototherapy used to treat various skin disorders, such as psoriasis or dermatitis (90, 91), we found no previous reference to how it might affect microbiome composition and diversity. The main drawback of using psoralen is its higher cost, being the most expensive among all the inactivation methods tested in this study.

The effect of heat, an affordable and broadly used method to inactivate highly pathogenic organisms, was also tested. By denaturing secondary structures of proteins, a broad range of enveloped and nonenveloped viruses can be inactivated with heat (37, 73, 76, 77, 92). The temperature and incubation times needed to render a virus noninfective vary depending on the particular agent, although, generally, incubation at higher temperatures reduces the time required for viral inactivation. However, by denaturing protein structures, heat may also alter the conformation of virion proteins and of other microorganisms present in the sample (76, 93). Thus, lower temperatures and longer incubation times may be preferred in order to preserve microbial structures. Here, we tested incubation at 56°C for 30 min, conditions that have been proven effective against a broad battery of viruses, including MERS-CoV, SARS-CoV, and SARS-CoV-2 (37, 39, 41, 77, 94). Results showed that such incubation conditions can be detrimental to bacterial communities in some cases. While stool and skin microbiome were mostly stable after heat inactivation, nasal samples suffered significant changes in their composition. In a recent study, heat inactivation of SARS-CoV-2 at 56°C for 30 min was able to preserve reverse transcription-quantitative PCR (qRT-PCR) sensitivity to different genes of the virus (39). In a different study, however, the same protocol was shown to reduce the levels of viral RNA detection by digital PCR (38). Due to its affordability and accessibility, heat inactivation can still be a good option when working with certain microbiome samples that do not involve further immunological tests, such as antigen detection or characterization. However, heat should be generally avoided in the processing of nasal samples, a finding of high relevance in the current COVID-19 pandemic.

In our study, we observed that skin and nasal samples’ bacterial communities were generally more sensitive to changes after inactivation. As discussed above, some of the approaches utilized can have detrimental effects on cell structure and nucleic acids present in the samples. Because these samples harbor low bacterial loads, there is a higher likelihood for overamplification of extraneous DNA despite rigorous quality control. On the other hand, and despite the higher loads of bacteria in stool samples, we observed that certain reagents, including ethanol, TRIzol, and formaldehyde, depleted some important intestinal commensals, such as *Coprococcus* or *Blautia*, while enriching others, such as *Bacteroides* or *Alistipes*. Our findings suggest that while some gut bacteria may be sensitive to specific inactivation reagents, the same reagents could improve DNA extraction of other taxa, likely due to differences in bacterial cell membrane structure. Oral samples seemed to be the most stable among those we tested. Some studies have previously reported a decrease in virucidal effectivity of certain viral inactivating agents depending on the protein content of the samples (37, 95). It is plausible that the high protein content in human saliva, including highly glycosylated mucins and proteins involved in exopolysaccharide synthesis, combined with the intricate interactions of oral bacteria with other carbohydrates and protein components of oral biofilms, could protect bacterial communities from the damaging effects of inactivation procedures (96–98).

Overall, and based on our results, we recommend the use of psoralen in combination with UV-A radiation as the inactivation agent that best preserves microbiome structure across most sample types, while we strongly discourage the use of 75% ethanol (Table 1). Although TRIzol did not introduce major changes to oral samples, it had a larger impact on samples from other body sites. In addition, inactivation with heat preserved stool and skin samples’ community structure better than other inactivation methods but caused considerable alterations in the nasal samples, which showed better results when inactivated with formaldehyde. Thus, the use of specific inactivation methods should generally be chosen based on the type of samples under study.

There are other factors that are often considered in inactivation studies, including the concentration of the inactivating reagent and incubation conditions, as well as the specific virus to be inactivated. For example, alcohol can effectively inactivate most enveloped viruses, although some nonenveloped viruses can persist (59, 99, 100). In the study by Schneider et al., a broad range of viral agents were inactivated with psoralen in combination with UV radiation, but the amount of exposure to UV required to reach inactivation varied across viruses (88). Similarly, different temperatures and incubation times yield variable inactivation results (37–39, 77, 94). Concentration of viral particles may also be a critical factor for inactivation efficiency (101–104). In the study by Pastorino et al. (102), temperature conditions generally considered virucidal and which can inactivate a broad range of viruses were not able to inactivate SARS-CoV-2 when samples contained viral loads greater than 6-log_10_ 50% tissue culture infective dose (TCID_50_). Viral inactivation methods may also depend on the type of assays to be performed: although next-generation sequencing does not generally require microbial cell structure integrity to be preserved, other microbiological and immunological analyses, such as serology or antigenic characterization of viruses, bacterial and viral coating with antibodies, as well as microscopy, do rely on cell integrity. In cases where molecular integrity of epitopes needs to be preserved, formaldehyde and psoralen treatments may be preferred (76, 88). It should be noted that our study only assessed the impact of inactivation in bacteria, and therefore, the potential effect on other fractions of the microbiome, such as fungal or viral communities, should be evaluated in future studies. In addition, bacterial contamination arising from laboratory reagents and kits can impact microbiome studies. When analyzing the abundance of contaminants across inactivation methods, we only identified one member of the *Bradyrhizobiaceae* family, which has been previously reported as a laboratory contaminant frequently found in DNA extraction kits and other reagents (105, 106). As we also identified this taxon in other groups, including untreated samples and samples treated with heat (which cannot be a source of microorganisms), it seems unlikely that contamination originated from the inactivating reagents. A more plausible alternative is that contaminants are introduced during regular sample processing, as previously shown (105–107). Finally, our samples were obtained from healthy subjects and do not contain infectious viruses. While the microbiome of infected patients is different from that of healthy controls (12–17), a comprehensive evaluation of all possible infection-associated microbiomes is beyond the scope of this work. Further, the use of healthy microbiomes to test the impact of storage condition or processing is well established in the literature (43, 46, 48, 108), and so, our study design follows a similar approach.

While our study did not exhaustively cover every possible inactivation agent and condition and our sample size per body site and inactivation treatment was moderate, our results clearly highlight the need to use appropriate treatments depending on the specific tissue being sampled. Larger studies using different inactivation conditions and sample types could uncover fine-grained interactions leading to the depletion or enrichment of taxa of interest. Nevertheless, we strongly encourage future studies to consider the guidelines here presented and, at a minimum, to test different inactivation protocols to ensure the robustness of their conclusions.

## MATERIALS AND METHODS

### Sample collection.

**FIG 5:**
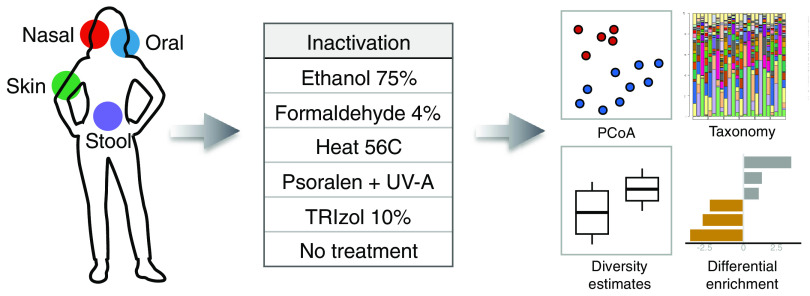
Study design. Forearm skin, oral, nasal, and stool specimens were obtained from five healthy subjects. Samples were aliquoted and treated with each of five inactivation methods plus an additional aliquot kept with no treatment. DNA was extracted, and 16S rRNA gene amplicons were sequenced using the Illumina MiSeq platform following standard protocols. Microbiome composition, diversity estimates, and differential enrichment analysis were performed to identify inactivation- and tissue-specific changes.

Five healthy volunteers participated in this study. Each of them donated a stool sample in a sterile container (109), and samples were aliquoted in approximately 100-mg aliquots and frozen within 2 h of collection. A trained member of the research team collected nasal (nares), oral mucosa (inner cheek), and forearm skin specimens in a clinical setting from volunteers using sterile swabs (catalog no. 220145; BD), and samples were immediately stored at −80°C. For each subject, six swabs were collected from the oral cavity, anterior nares, and forearm skin, and six stool aliquots were made, for a total of *n* = 120 samples (Fig. 5). Sample collection for the research was approved by the institutional review board (IRB) at The Icahn School of Medicine at Mount Sinai in New York, and all subjects signed informed consent.

### Inactivation protocols.

Specimens from each subject were treated with each of the following five inactivation methods: heat (39), ethanol (71), psoralen (88), TRIzol (110), and formaldehyde (79), as well as a no-treatment method consisting of immediate thawing and DNA extraction. Heat-treated samples were suspended in sterile PBS and incubated for 30 min at 56°C in a heat block (Denville Scientific). Absolute ethyl alcohol (ethanol) was diluted with PBS and added at a 75% final concentration, and samples were incubated at room temperature for 30 min. Psoralen (≥99%; Sigma-Aldrich) was added at a final concentration of 10 μg/ml in dimethyl sulfoxide (DMSO); samples were incubated at room temperature for 30 min and then transferred to a 24-well plate and exposed to UV radiation in a BioDoc-It imaging system (UVP) for 30 min. TRIzol reagent (Invitrogen) was added at a final concentration of 10% in sterile PBS, and samples were incubated at room temperature for 10 min. Formaldehyde was added at a final 4% concentration in PBS, and samples were incubated at room temperature for 30 min (Fig. 5).

### DNA extraction and 16S rRNA gene library construction.

On the same day that samples were inactivated, DNA was extracted with a DNeasy PowerLyzer PowerSoil kit (Qiagen) following the manufacturer’s instructions. The concentration of extracted DNA was estimated by using a NanoDrop ND-1000 spectrophotometer (Thermo Scientific). For each sample, the V4 region of the 16S rRNA gene was amplified in triplicate, following the protocol from Caporaso et al. (111). The amplified replicates were pooled, their DNA concentration was measured using Qubit fluorometric quantitation, and sequencing was performed in an Illumina MiSeq platform (paired-end 250 bp), as previously described (111–113), at the Genomics Core at NYU (New York). This resulted in a total of 128 samples, including 8 blanks (6 DNA extraction controls, treated with each inactivation method or no treatment, and 2 PCR controls).

### 16S rRNA gene sequencing data analysis.

The resulting raw sequencing reads were initially analyzed using QIIME2 v2020.8 (https://qiime2.org) (64). Reads were demultiplexed and quality filtered, and pair ends were joined with DADA2. Filtered sequences were clustered and assigned to amplicon sequence variants (ASVs) using the GreenGenes v13.8 full-length sequence database (114). Common reagent bacterial contaminants were removed following recommendations from Salter et al. (105). The list of removed contaminants can be found in Table S1 in the supplemental material. After filtering, a mean ± SD of 45,519 ± 33,708 sequences per sample remained, with a mean of 84.42 ± 58 observed features per sample. One oral sample inactivated by heat yielded less than 500 reads and was eliminated from the data set. A phylogenetic tree was generated using the align-to-tree-mafft-fasttree function in QIIME2 and was used to calculate phylogeny-informed distances between samples (UniFrac distances). Alpha- and beta-diversity indices were measured on tables rarefied at 4,385 sequences per sample. All other statistical analyses were performed on R software v4.0.3 (115). Principal coordinate analysis (PCoA) and density plots based on UniFrac distances were visualized with the ggplot2 (116) package in R, and a nested permutational multivariate analysis of variance (adonis) test was applied to compare the effect size of inactivation methods and body site on beta diversity, stratifying per subject, using the adonis function from the vegan package in R with 999 permutations. To test for homogeneity of variability (dispersion) among inactivation methods and body sites, permutation multivariate analysis of dispersion was conducted with the betadisper and permutest functions, also from the vegan package. Bacterial relative abundances were summarized in barplots, using the barplot package. Linear models for alpha diversity were constructed using the glm function in the stats package in R. We used an identity link function and a Gaussian error model in accordance with the apparent distribution of the data. The input formula contains linear terms and an interaction term between body site and treatment as follows: SE (Shannon entropy) or OF (observed features) ∼ subject + body + treatment + body/treatment. Coefficients with *P* values of <0.05 were kept in the model. Differences in composition at the phylum level were calculated as the ratio of the difference between bacterial relative abundances in each treatment compared to no treatment (fold change). Linear discriminant analysis effect size (LefSe) (55) was used to identify differentially abundant taxa between each treatment compared to no treatment for each sample type. Kruskal-Wallis and pairwise Wilcoxon tests were used to compare differences between inactivation protocols and body sites. For all statistical analyses, a *P* value of <0.05 was considered significant. ANCOM analysis (56), as implemented in QIIME2, was applied to compare microbial taxonomic differences between blank controls treated with each inactivation method and no treatment.

### Data availability.

Raw sequencing data are available from the NCBI Sequence Read Archive under the BioProject accession no. PRJNA724050.
