# Supplementary figures and images for: Viral Inactivation Impacts Microbiome Estimates in a Tissue-Specific Manner
Source: mSystems. 2021 Oct 5;6(5):e00674-21. doi: 10.1128/mSystems.00674-21 (PMC8547476; doi:10.1128/mSystems.00674-21)

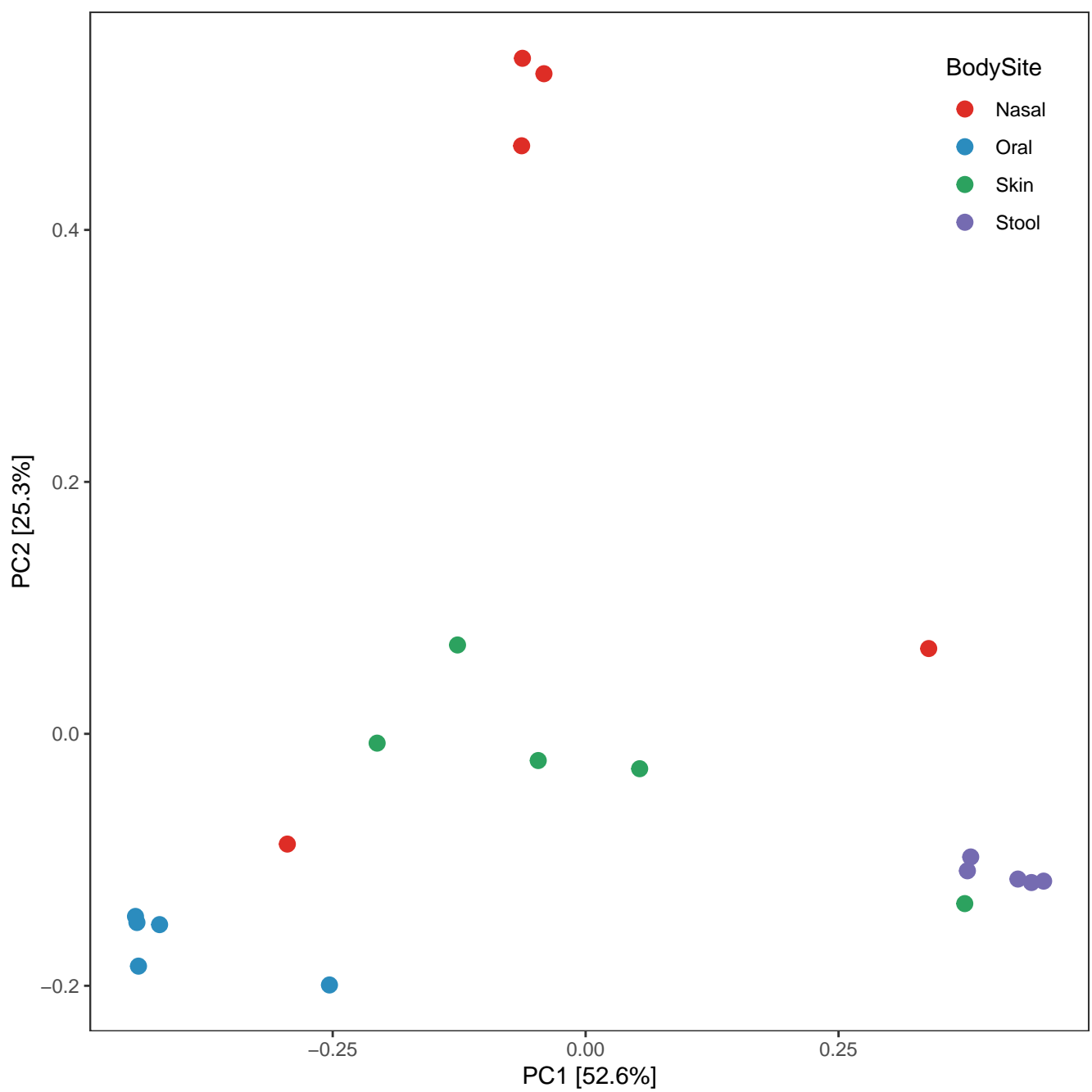

Supplement: FIG S1 [file msystems.00674-21-sf001.pdf]

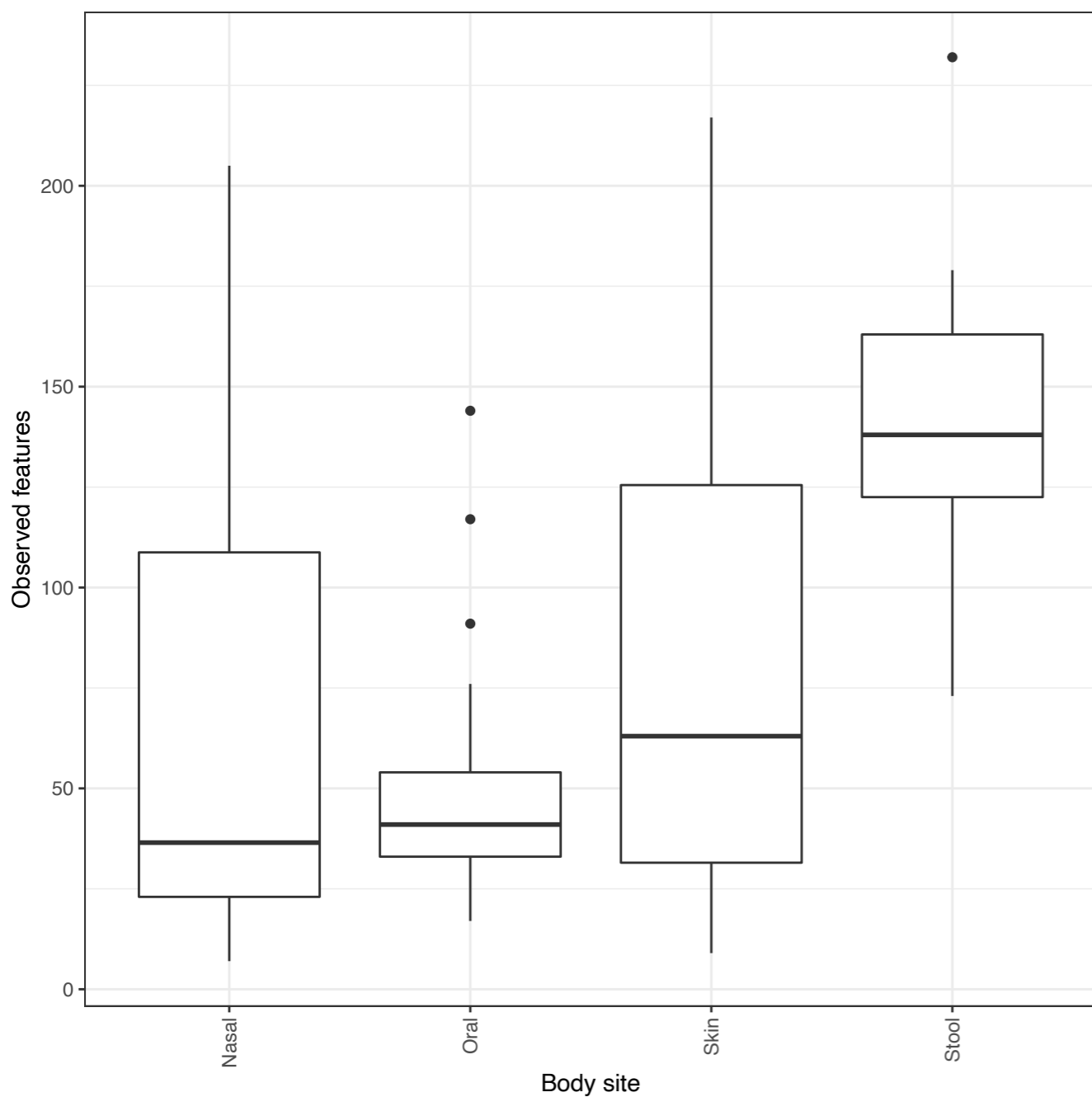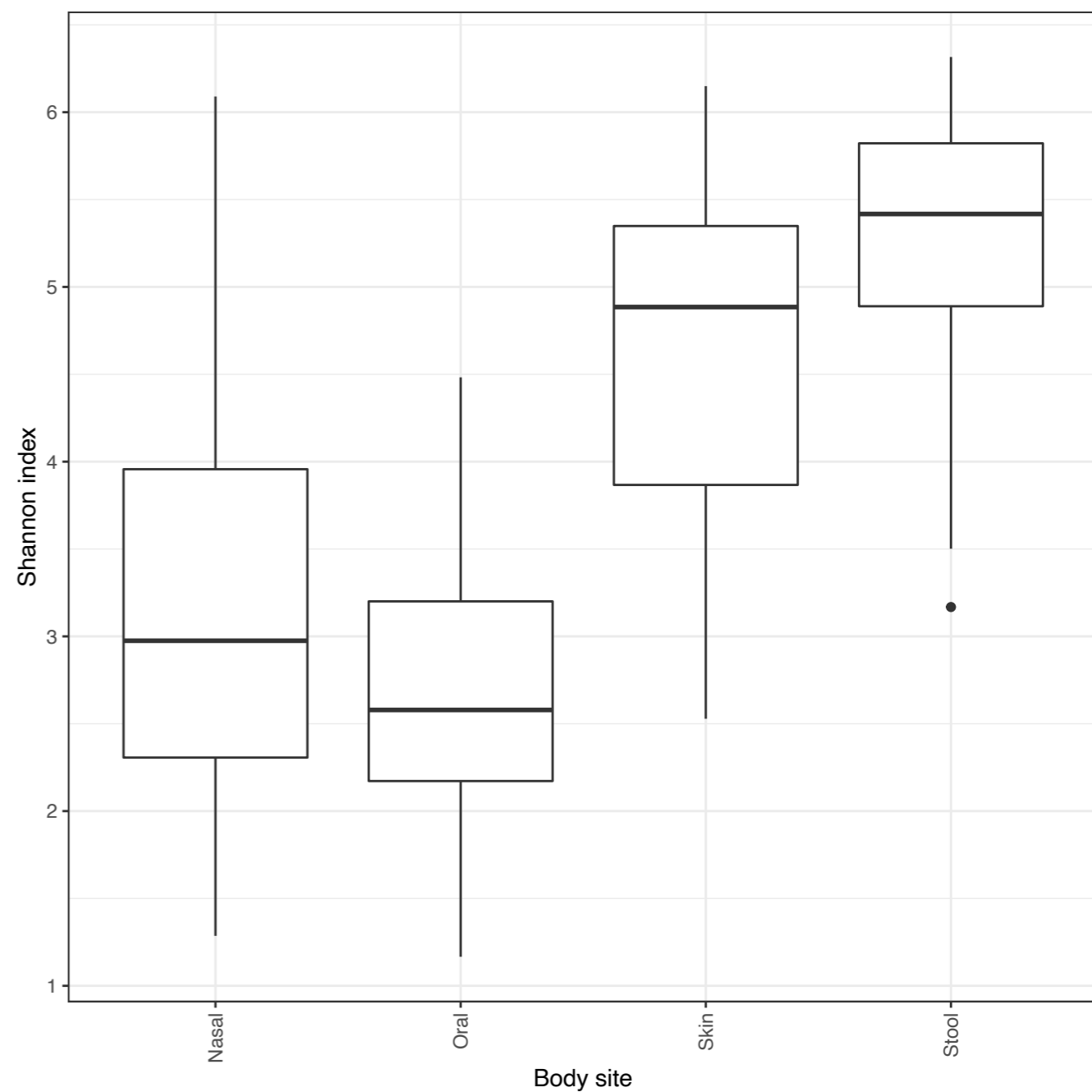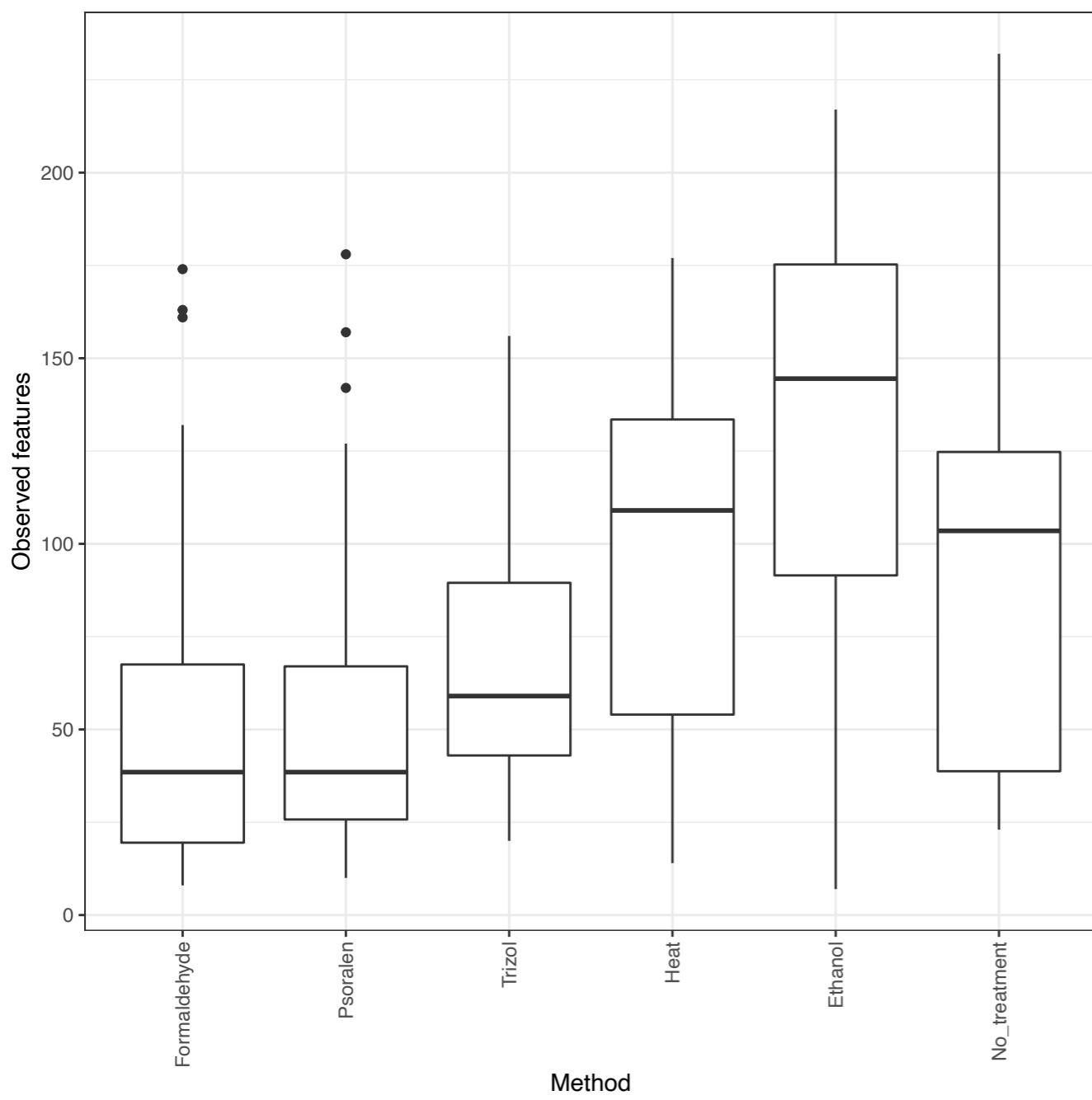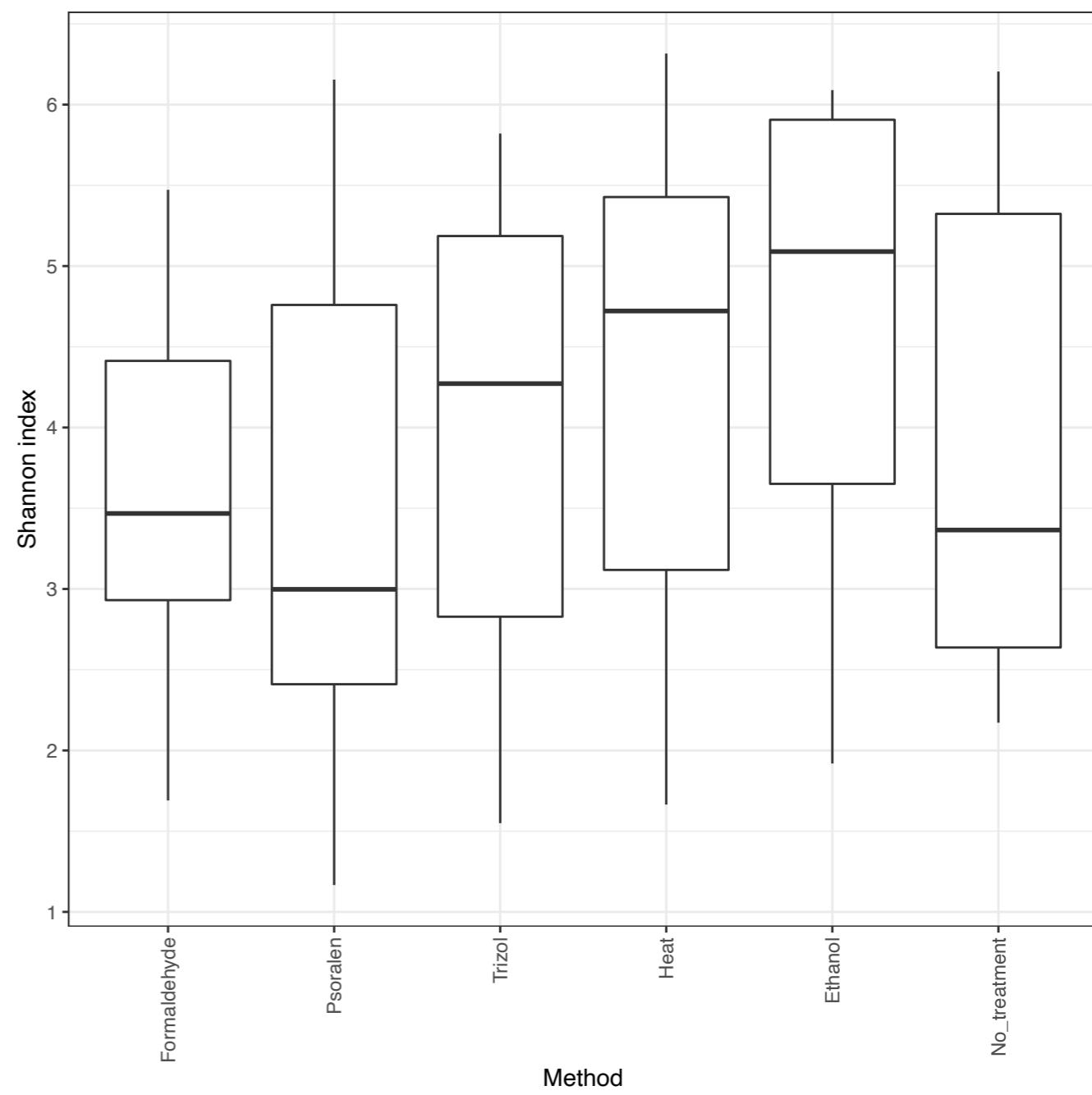

Supplement: FIG S2 [file msystems.00674-21-sf002.pdf]

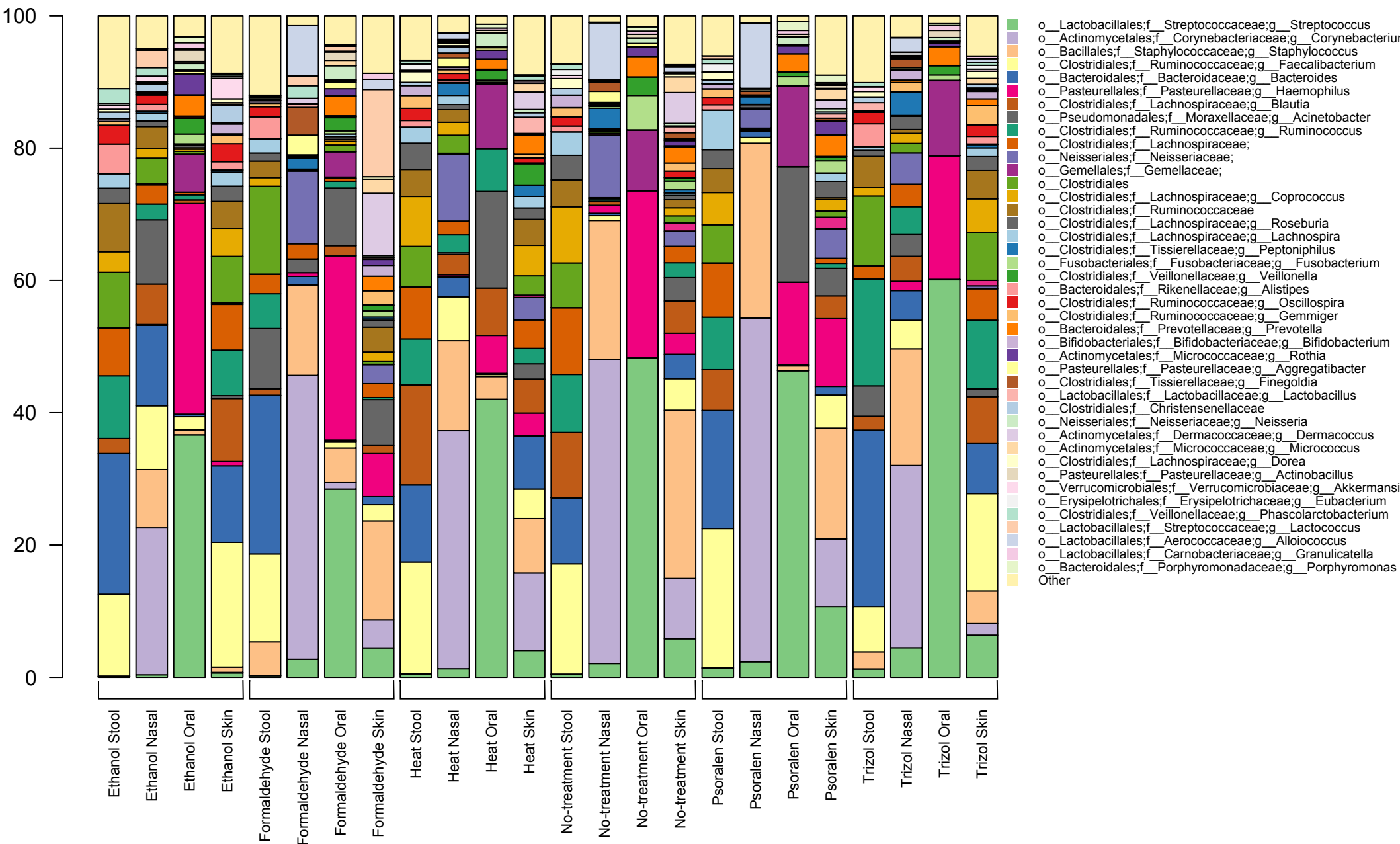

Supplement: FIG S3 [file msystems.00674-21-sf003.pdf]

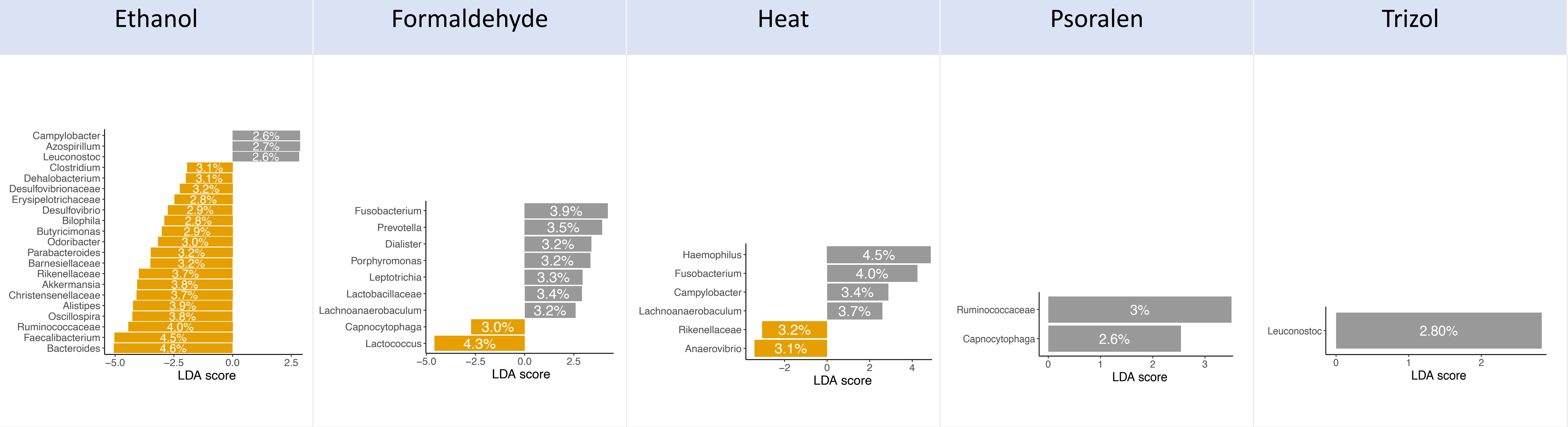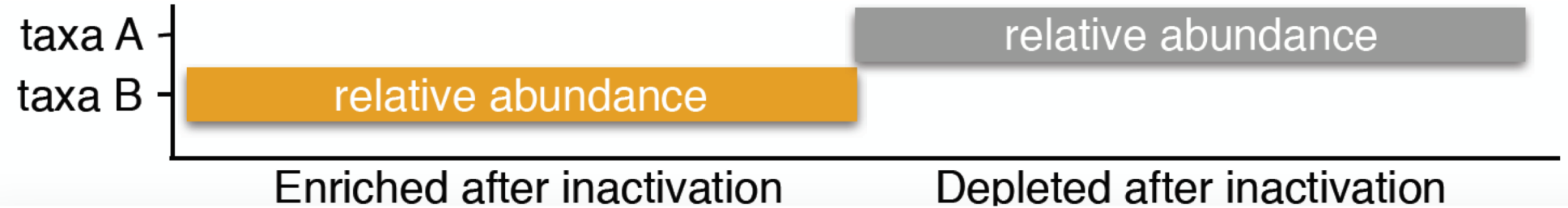

Supplement: FIG S4 [file msystems.00674-21-sf004.pdf]
