# Supplementary material for: Viral Inactivation Impacts Microbiome Estimates in a Tissue-Specific Manner
Source: mSystems. 2021 Oct 5;6(5):e00674-21. doi: 10.1128/mSystems.00674-21 (PMC8547476; doi:10.1128/mSystems.00674-21)
Supplement: FIG S5 [file msystems.00674-21-sf005.pdf]

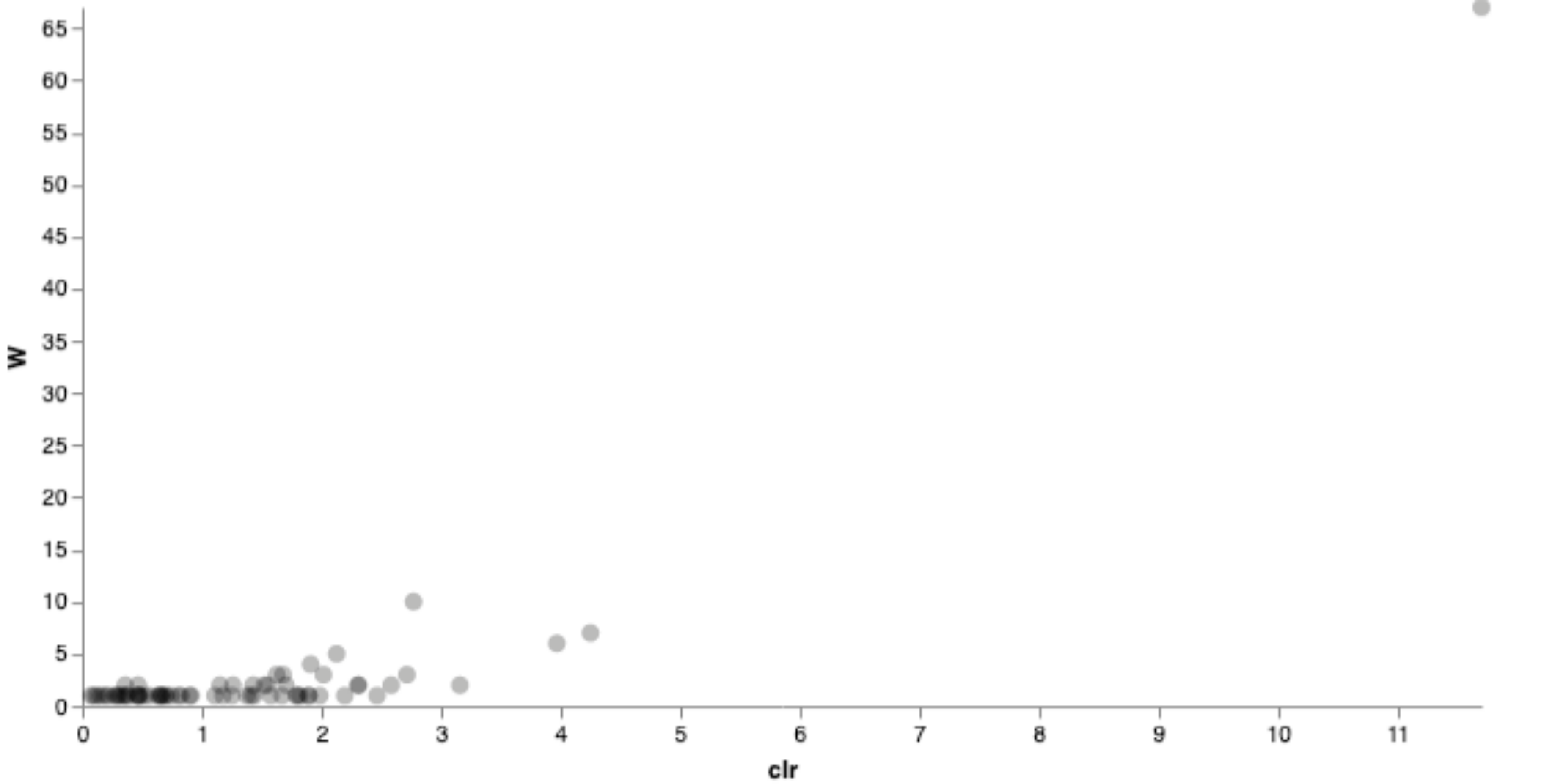

ANCOM statistical results

	W
**k__Bacteria; p__Proteobacteria; c__Alphaproteobacteria; o__Rhizobiales; f__Bradyrhizobiaceae**	67
